# An Indoor Positioning System Based on Wearables for Ambient-Assisted Living

**DOI:** 10.3390/s17010036

**Published:** 2016-12-25

**Authors:** Óscar Belmonte-Fernández, Adrian Puertas-Cabedo, Joaquín Torres-Sospedra, Raúl Montoliu-Colás, Sergi Trilles-Oliver

**Affiliations:** 1 Institute of New Imaging Technologies (INIT), Jaume I University, Av. Vicente Sos Baynat s/n, 12071 Castelló de la Plana, Spain; jtorres@uji.es (J.T.-S.); montoliu@uji.es (R.M.-C.); strilles@uji.es (S.T.-O.); 2 Soluciones Cuatroochenta S.L., Av. Vicente Sos Baynat s/n, Espaitec2 Building, 12071 Castelló de la Plana, Spain; adrian.puertas@cuatroochenta.com

**Keywords:** Ambient-Assisted Living (AAL), indoor positioning, Machine Learning, Message Queuing Telemetry Transport (MQTT) connectivity protocol

## Abstract

The urban population is growing at such a rate that by 2050 it is estimated that 84% of the world’s population will live in cities, with flats being the most common living place. Moreover, WiFi technology is present in most developed country urban areas, with a quick growth in developing countries. New Ambient-Assisted Living applications will be developed in the near future having user positioning as ground technology: elderly tele-care, energy consumption, security and the like are strongly based on indoor positioning information. We present an indoor positioning system for wearable devices based on WiFi fingerprinting. Smart-watch wearable devices are used to acquire the WiFi strength signals of the surrounding Wireless Access Points used to build an ensemble of Machine Learning classification algorithms. Once built, the ensemble algorithm is used to locate a user based on the WiFi strength signals provided by the wearable device. Experimental results for five different urban flats are reported, showing that the system is robust and reliable enough for locating a user at room level into his/her home. Another interesting characteristic of the presented system is that it does not require deployment of any infrastructure, and it is unobtrusive, the only device required for it to work is a smart-watch.

## 1. Introduction

The number of older people in the world will exceed the number of young people by 2050, according to United Nation Population Division of the Department of Economic and Social Affairs [1]. The percentage of older people is projected to reach 21% in 2050, which will reach one third of the population in the case of developed countries. Another key finding is that the life expectancy is projected to rise from 70 years in 2010–2015 to 77 years in 2045–2050. Finally, as a remarkable example, fertility in all European countries is 1.6, clearly below the level required for full replacement of population in the long run (2.1 children per woman, on average) [2].

Another key prospect is the urban population increment, which is expected to be 84% of the total population by 2050 [2], this growth being faster in developing countries than in the developed ones. In contrast, the rural population is expected to decrease slowly after 2020. The final result at that time will be that elder population in urban areas is getting older, due to two main factors: the decline in the birth rate and the population movements from rural to urban areas.

Several studies have shown that elderly people want to live at home as long as possible [3,4]. The Center for Disease Control and Prevention defines Aging in Place (AIP) as the ability to live in one’s own home and community safely, independently, and comfortably, regardless of age, income, or ability level [5]. A short term challenge in developed countries and a medium-term challenge in developing countries is represented by elderly people living in home. Information Technologies have shown to be an effective tool to improve management of chronic or noncommunicable diseases [6]. In particular, they have proven as a very useful tool for ill people living at home [7].

Caregivers are mainly family and friends which cause loss of productivity and income, as well as stress-related illnesses [8]. There is a short-term impact on jobs of families and caregivers of elder people and noncommunicable diseases patients, as well as a long-term impact in their career and retirement savings. As an example, it has been estimated that unpaid caregivers provide 17.5 billion hours which can be valued in terms of money as much as 216.5 billion dollars in 2012 (https://www.genworth.com/cost-of-care/landing.html) in the USA.

In-home monitoring systems have become a valuable tool for elder people to live in their own homes while providing supportive services for health-care illness prevention. In [9] a system classification for in-home monitoring systems was presented, in most of the systems analyzed, health-care practitioners, families and friends and caregivers are the main consumers of the data provided by the monitoring system, instead of the individual being monitored.

Most of the in-home monitoring systems already presented are based on ad-hoc sensor networks deployed at home. The main sensor types used in these networks range from inexpensive passive infra-red motion (PIR) sensors to expensive video cameras. Some of these technologies are seen as obtrusive, specially in the case of video cameras. Moreover, such solutions, based on ad-hoc hardware deployment are seen as uncomfortable, impractical and expensive in some cases.

**Figure 1:**
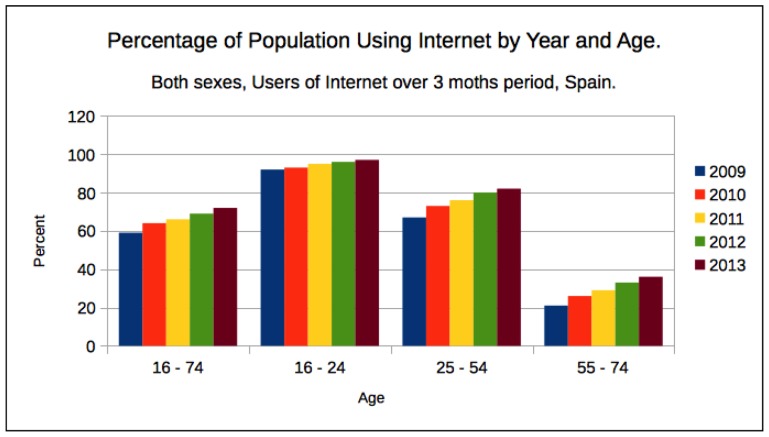
Percentage of Spanish population, both sexes, using Internet by year and age, over 3 moths period. First column shows total for any age within 16–74 years.

Figure 1 shows the growth in the use of Internet connection by year and age group, it can be seen that more elderly people are using Internet at home, and most of them are using Wireless Access Points (WAPs) for this purpose. The current trend is that elderly people are adopting the use of smartphones to keep in contact with relatives and friends. Current data provided by Eurostat (http://epp.eurostat.ec.europa.eu/portal/page/portal/eurostat/home) states that 64.5% of the population in Spain live in flats, 21.1% in attached houses, and 20% of the population between 65 and 74 years old, retired or inactive, access to the Internet using mobile devices.

In this paper, we present a robust, unobtrusive, inexpensive and easy to use in-home monitoring system based on WiFi fingerprints registered by a smart-watch. Although there are other technologies available for in-home monitoring, such as bracelets and smart-phones, smart-watches has the advantage of being a highly non-obtrusive, cheap and easy to use tool, which can also provide direct communication between the user and care-givers, nurses or general practitioners.

The rest of the paper is structured as follows, Section 2 presents the related work, a characterization based on eight items is given and used to classify the reviewed works. Section 3 presents the AwareIndoorLoc system, its different parts, how they communicate to each other and the technologies used for developing them. The system operation is described in Section 4. The evaluation of the systems through experiments in real scenarios is presented in Section 5, and the results discussed in Section 6. Finally, Section 7 presents the conclusions and the future work.

## 2. Related Work

This section firstly reviews the related work on Indoor Positioning Systems (IPS), paying special attention to WiFi-based fingerprinting positioning. Secondly, some related works on AAL are reviewed and compared. Finally, the main characteristics of our WiFi-based positioning system for AAL are presented.

### 2.1. Indoor Positioning Systems

Although there are some surveys which classify IPS [10,11,12], these systems can be broadly classified into two main groups: those technologies that needs some infrastructure deployment, and those which do not need any infrastructure deployment, such as, for example, systems based on magnetic field fingerprinting [13,14]. In the same way, those technologies which need some infrastructure deployment can be subdivided in those which needs an ad-hoc deployment, for example systems based on Bluetooth Low Energy beacons (BLE) [15]; and those which take profit of already deployed infrastructures, for example systems based on WiFi fingerprinting [12]. An extensive review of Indoor Positioning Systems (IPS) can be found in [16].

WiFi-based Fingerprint positioning systems are based on the Received Signal Strength Indicator (RSSI) level from the Wireless Access Points (WAPs). This technique is based on the fact that the signal loss due to path and penetration losses, reflection and refraction effects is complex enough to be modeled as a function of location. Instead, WiFi fingerprinting assumes that a signal mapping exists and that such a map can be reconstructed measuring the RSSI signal at discrete locations of the mapped area. WiFi fingerprinting consists of two stages, in the first stage, for each mapped position $x_{i}$ a vector of all detected RSSI signals $S={\left[S_{i1},S_{12},...,S_{in_{W}}\right]}^{T}$, where $n_{W}$ is the number of total WAPs present in the whole mapping area. The pairs of all measures and the vector of RSSI signals $\left(x_{i},S_{i}\right)$ is the WiFi fingerprint database. In a second stage or system operation, a vector of RSSI signals is measured $\hat{S}={\left[{\hat{S}}_{i1},{\hat{S}}_{12},...,{\hat{S}}_{in_{W}}\right]}^{T}$ and compared, using some similarity metrics, with the RSSI vectors in the database. In its basics form, the location assigned to the vector $\hat{S}$ is that which provides the minimum distance for the chosen metrics [17], namely $\hat{x}=\{x_{i}|min(∥d\left(\hat{S},S_{i}\right)∥)\}$. In reference [18] the authors present a complete analysis of distance and similarity measures for WiFi fingerprinting for indoor positioning system.

Instead of storing a position $x_{i}$ for each vector of RSSI signals $S_{i}$ in the database, a class $c_{i}\in \left\{c_{1},c_{2},...,c_{n}\right\}$ where *n* is the total number of different classes, can be associated with each vector $S_{i}$, in this case the fingerprinting database can be used to solve a classification problem: given a vector of signal $\hat{S}$ an estimate of the class $\hat{c}$ should be provided. In this paper the latter representation has been used, where classes are the rooms in an apartment, for example $c_{i}\in \left\{Kitchen,Living-room,Bedroom,Bathroom\right\}$.

One of the major advantages of the methods based on WiFi fingerprints is that they rely on the already existing WiFi infrastructure. Therefore, the location of the user can be obtained without deploying any additional infrastructures. However, WiFi was not designed as bases for indoor positioning. Taking into account the existing obstacles introduced by the indoor environment (including reflections and multi path interference) the spread of radio signal in indoor environments is very hard to predict [19]. In addition, in WiFi-based positioning systems, the user typically carries the device with him. His motion or how the device is carried are an important factor that affects the measured RSSI values [20]. For an extensive study and comparison of different IPS based on WiFi fingerprinting the reader can review [12].

### 2.2. Indoor Positioning Systems in Ambient-Assisted Living

There are papers that review the works presented in the field of AAL, some of the reviews focus on works directly related with fall detection, while others focus on wearable sensor based systems. Axisa et al. [21] present a review of smart clothing technologies for health-care, illness prevention and citizen medicine. These technologies are based on devices directly attached to the human body and are able to measure some physical constants as skin temperature and conductivity. Some of the reviewed solutions can also determine the location of the user inside a building and to use Global System for Mobile communications (GSM) smartphone networks to notify alarms. It is worth noting that most of the reviewed works in this paper are proprietary solutions.

User requirements for wearable health monitoring sensors are analyzed in reference [9]. From the authors point of view, the most challenging points when developing wearable sensors for in home health monitoring are: reliability and robustness, unobtrusiveness, user identification, communication, zero maintenance and fault recovery. Communication approaches are reviewed in detail as they play a central role in the independence of living in a home.

A survey of fall detection methods is presented in reference [22]. These methods can be grouped into three main categories depending on the technology used: wearable device, ambiance device and camera-based. All of these methods have multiple drawbacks and require the user to acquire some special equipment or accessory, such as wearable sensors or video cameras.

Taking into account the previous classification, the most representative works are commented upon. To recall, the ambiance device approach is to use a variety of sensors installed in the house. These sensors detect when a person is close enough and, therefore, detect the location of the person. In reference [23], InfraRed (IR) sensors are used to monitor the presence in rooms with no doors, while magnetic switch sensors are used in rooms with doors. Falls are detected using an ad-hoc sensor which bases its operation on the data fusion coming from three different sensors: accelerometer, tilt and vibration sensors. All information is transmitted using RF signals to an in-home or remote processor unit. IR and magnetic switch sensors are cheap, but the ad-hoc sensor to detect falls could be expensive. Communications are performed using RF, so some adapter device would be needed to interconnect them to the Internet. Demongeot et al. present in reference [24] a system to monitor patients at home. Passive InfraRed (PIR) sensors attached to each room are used for patient location, generic accelerometer sensors are used to detect falls, and specific sensor are used to measure the respiratory rhythm, blood pressure and cardiac parameters. Communications between sensors and a processor unit are established by means of the Controller Area Network (CAN) [25]. XML format is used to transmit and store data, but any other neutral language could be used, for example json or plain text. Data is taken each hour, and used to detect deviation from a predefined behavior which, in case it occurs, will fire some alerts. In reference [26] a method for helping in the medicine intake management task is presented. This system checks the possible conflicts in the prescribed medicines of elderly people. The medicines information registration is automated using an RFID card. Additionally, the data of the system can feed other subsystems in a Smart House. The work in reference [27] describes a flexible floor-based indoor positioning system. This system is based on capacitive sensors that are specifically designed to detect position and potential falls of users at home. They use passive floor mats of a rectangular shape equipped with active sensor elements on two adjacent outer sides. In reference [28], the authors have developed the n-Core Polaris system. This system defines a sensor network using Zigbee as communication protocol. The sensor network comprises tags, readers and sensor controllers. A web server connects to the readers and offers the position information to a wide range of possible client interfaces. A database is created to register historical data, such as alerts and location tracking. In reference [29], the authors present the iLoc sytem, which uses an ultrasound ranging for indoor positioning. This system comprises badges, detector nodes (located at known fixed positions) and a server running the positioning algorithm. The badge is an ultrasound transmitter which emits ultrasound pulses. The detector nodes receive these pulses and send the reception times to the server. The server calculates badge position. The work in reference [30] presents a method for indoor positioning using microwave signals. For this purpose, beacons and transponders are used. The beacons are located at some reference points. The transponders are attached to people’s objects. Each beacon selects the low frequency difference signals. Based on these measurements the distances to transponders are calculated. In reference [31] a commercial indoor location system is used to infer models about user movements in a nursing home for allowing experts to understand individual behavior. The system uses bracelets periodically connected to beacons using the ZigBee protocol, which in turn are connected to servers using Ethernet protocols. All data collected is analyzed using data-mining techniques to find out user’s behavior, such as preferred location, and how this behavior changes over time. Virtual Butler [32] is a location-aware human-machine interaction system which provides the interface between the elderly and the smart infrastructures. Passive Infrared sensors are used to detect the presence of the user in a room, in addition a bed presence is used to detect the user in bed. Based on the user location and an agent based architecture the system is able to answer questions made in natural language. A publish/subscribe protocol is used to interchange data between the agents in the system.

Some other works in the AAL field use some kind of wearable device plus a camera device. A smart home based health for monitoring diabetes patients is presented in reference [33]. The system is able to integrate data coming from wearable sensor, environmental sensor (temperature, humidity, light, etc.) and cameras. Wireless communications are securely provided by means of Internet services. Analytic is used on the raw gathered data to recognize the activity of the monitored patient, and to give feedback to the patient in form of recommendations about food habits. The work presented in reference [34] uses different data sources to note whether a person falls. These data sources consist of motion information, audio stream and video images. The data are obtained from wearable device such as overhead cameras, microphone arrays and body sensors. The analysis of this data can detects a fall. Furthermore, this system estimates the severity of the fall monitoring the data after the fall. It uses a ontology and rule-based evaluation for this purpose. Ozcan et al. [35] propose another video camera-based approach for fall, sit and lying down detection. In this system, the user wear a CITRIC camera and the computation is performed by means of an embedded microprocessor. The image analysis method is based on the oriented image gradients, which takes into account the orientation of the camera frame during a fall.

A different approach is proposed in reference [36]. Owl Positioning System (OwlPS) is a WiFi based system for indoor positioning. This system is composed of a smartphone, some WAPs, an aggregation server and a positioning server. The smartphone sends different packages and each WAP extracts the corresponding RSSI. Next, the aggregation and position server collects and analyses the packets and obtain the position of the smartphone.

In order to compare the formerly reviewed works, we propose the following characteristics as they well describe them in the context of AAL:

- *Sensor Type*: refers to the type or types of sensors used for ambient monitoring.
- *Cost*: the cost in terms of money and efforts to deploy the monitoring system. Scale: Expensive, Average, Cheap, Inexpensive.
- *Scalability*: how easy it is to add new rooms/ambients to already monitored areas. Scale: High, Medium, Low.
- *Obtrusive*: the feelings of the users about their privacy invasion: High, Medium, Low.
- *Connection*: how the sensors are connected with the monitoring system. Scale: WiFi, Zigbee, Ultrasound, others.
- *Interoperable*: could be the system described interconnected with other monitoring systems: glucose, heartbeat, and others. Scale: Yes/No.
- *Extensible*: can be the system used in other kind of monitoring use. Scale: Yes/No.

Table 1 shows the classification of works reviewed according the above characteristics. The work in reference [36] is the only one that uses WiFi capabilities present in a mobile phone for positioning a user. On the contrary, WiFi capabilities present in a smart-watch attached to the wrist of the user is used in our work. No new infrastructures are required for using our system, so the only cost is the acquisition price of the smart-watch, in contrast with systems where deploying some infrastructures is needed, for example those relying on cameras [33,34], beacons [28,30,31], or infrared sensors [23,24,32]. Most of the analyzed, including the presented work, scale well with the only exception of [27] based on capacitive sensors. Obtrusive issues are mainly associated to the use of camera [33,34,35], our system is unobtrusive, the user has only to wear a smart-watch which can be used as a simple watch. There is a great diversity in the connection used for each reviewed system, in the presented work WiFi connectivity is used. Most of the reviewed works are interoperable with other monitoring systems. With the exception of [27] that uses sensors specifically designed, our work makes use of standards whenever possible, for example in the case of the MQTT connectivity protocol. In the same way, most reviewed works are extensible, so they can be applied to other uses cases, with the only exception of [26,27], the presented work could be easily used in any other where location will be a key aspect. The system presented in reference [32] and ours use a publish/subscribe protocol, which is focused on Machine to Machine communications.

## 3. *AwareIndoorLoc* System Description

This section presents first the theoretical methods and procedures used in the present work. Then, the three parts our system is composed of are presented: the wearable device and the application deployed on it, and the back-end system, which in turn is composed of the two parts, the system in charge of the data collection and administration, and the web-based management application. The interaction between each of these sub-systems is presented too.

### 3.1. Theoretical Methods and Procedures

To model WiFi signal propagation is a complex task due to path loss, penetration loss in the walls, ceiling, floor and other constructive elements and furniture, reflections and diffraction in these same elements, and body absorption. Mapping fingerprinting assumes that an RSSI map exists and it is constructed by measuring the RSSI at some locations, and uses this map as a database for location purposes. To find out the current location of a user at room level based on the RSSI mapping can be seen as a classification problem where each class represents a different room. Given a vector of RSSI measures at current position of a user $\hat{S_{i}}={\left[{\hat{S}}_{i,1},{\hat{S}}_{i,2},...,{\hat{S}}_{i,n_{W}}\right]}^{T}$ a location class $c_{i}\in \left\{Room_{1},Room_{2},...,Room_{N}\right\}$, where *N* is the total number of classes present in the database, should be estimated.

In the present work five well known supervised Machine Learning classification algorithms were used. In supervised learning, a set of training data, in this case a set of pairs $\left(S_{i},c_{i}\right)$ is used to train the algorithm. Then another set of pairs $\left(S_{i}^{\prime },c_{i}^{\prime }\right)$, called the testing database, can be used to asses the performance of the built classifier. The assessment consist on comparing the result provided by the classifier $\hat{c_{i}^{\prime }}$ for each input vector in the testing database $S_{i}^{\prime }$ with the real class $c_{i}^{\prime }$.

To provide a new localization method is not an objective of the present work. Instead, well known classification algorithms were used. The five Machine Learning algorithms used in this work and their configuration were:

- Multilayer Perceptron [37], a neural network algorithm. Multilayer Perceptrons can be used as classification algorithms. In the present case, the neurons in the output layer corresponds with each mapped room present in the training database, and the neurons in the input layers corresponds with the Signal Strength measured for each WAPs present. The number of neurons in the hidden layer has been set to $(N_{WAPs}+N_{labels})/2$, where $N_{WAPs}$ is the total number of WAPs present and $N_{labels}$ is the number of labels.
- Support Vector Machine [38], a geometric based classification algorithm. A linear kernel was used in our case, linear kernels split the features space in disjoint regions using hyper-planes. In this work each disjoint region limited by hyper-planes represent a mapped room present in the training database.
- Decission Tress, C4.5 [39]. In a decision tree, each internal node represent a test on an attribute (the RSSI for a particular WAP in our case), and each branch an outcome of the test. The leaf nodes represents a class label. A binary-tree representation was used in our case, each internal node represents a true/false test on the RSSI intensity for a particular WAP of the type: is the intensity level greater than some level?, if the answer is true the corresponding true branch of the binary-tree is followed to a new node. Each leaf node corresponds with a mapped room present in the training database.
- Random Forest [40], ensemble classification algorithm. The number trees used in the ensemble was set to 100, which has empirically proven to provide good results for this particular training data. Each leaf node corresponds with a mapped room present in the training database.
- Bayesian Networks [41], statistics based classification algorithm. A Bayesian Network is an Acyclic Directed Graph (ADG) where each node represents a state and each arch a transition between states with certain probability. In our case, the estimates for these probabilities were calculated from data.

An ensemble classifiers combining the result of the five presented classification algorithms were also used. This ensemble classifier works as follows: for each input RSSI vector, each of the five classifiers estimates a room as the current position of the user, which in turn adds a vote for that room. The output of the ensemble classifier is the room which receives more votes, in case of ties, the estimated room with less uncertainty is provided as final output.

### 3.2. Overall System Description

**Figure 2:**
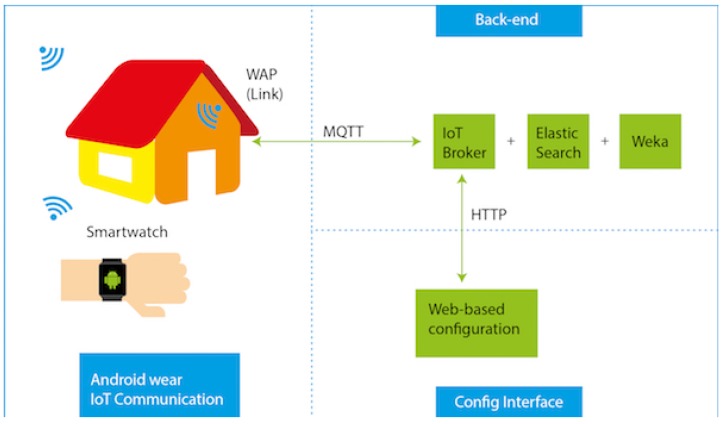
The three parts of the proposed system.

The proposed system is composed of two main parts: first, a mobile application which runs in an Android Wear device and communicates with the back-end system; second, a back-end system which contains a system in charge of creating the positioning models based on Machine Learning algorithms. The back-end system also contains a web-based configuration interface to manage the different devices and users that the system can manage at same time. Figure 2 shows a top level view of the system design.

The communications between the wearable and the back-end system were based on the MQTT connectivity protocol [42]. This protocol is based on the TCP/IP protocols (Although, there is a non-TCP/IP version named MQTT-SN focus on technologies such as Zigbee.) and is focused on Machine-To-Machine (M2M) connectivity for limited-resources devices on unreliable networks, with very low footprint. The MQTT connectivity protocol is based on the publish/subscribe paradigm [43], producers publish messages in the context of some topics, and clients subscribe to such topics. MQTT supports three different levels of Quality of Service (QoS): for QoS = 0 a client would receive a message at most once, but the client could not receive some messages; for QoS = 1 a client would receive a message at least once, with possible duplicate messages; for QoS = 2 the client will receive a message exactly once, no message would be lost.

### 3.3. Wearable Device

One of the most popular wearable devices is the smart-watch. In part, its popularity comes down to the fact that it can be seen as an extension of a smart-phone, which, at the same time, is its main drawback: why do we need another device that it is just an extension of a smart-phone? From our point of view, its main advantage is to be a regular device, it looks like a common watch easy to wear and use. Another advantage with regards to the smart-phone is that a smart-watch is always attached to the user, roughly speaking, one is less likely to forget a smart-watch on top of the beside table that a smart-phone.

The wearable device used was a Smart Watch 3 by Sony. This particular smart-watch runs Android Wear 6 version and embodies a WiFi chip that is accessible through Android API, and GPS, accelerometer, compass, gyroscope and ambient light sensors. Connectivity is supported through WiFi, NFC and Bluetooth. The resolution of its 1.8” screen is 320 × 320 pixels, and it costs less than 150 $ at the time of this writing.

#### 3.3.1. Smart-Watch Device Characteristics

Most smart-watches embody several sensors like accelerometer, gyroscope, ambient light intensity, compass, and so on. On the connectivity layer, most of them also embody Bluetooth, NFC and WiFi communications. Current Android Wear APIs allow the access to the WiFi chip, making it possible to use WiFi fingerprinting technology as a suitable positioning candidate to be deployed in such devices.

Another important characteristic of these devices is their energy consumption. For all different existing smart-watch models, one charge of battery lasts a whole day, even with the wifi chip switched on. This is a minimum requirement for monitoring applications, the user needs to charge these devices only once a day, which, on average, is the common battery duration for most smart-phones.

Finally, most of these devices also include a GPS chip, so our system could be combined with GPS to provide the location of the user outdoors and indoors.

#### 3.3.2. Android Wear and IoT Communications

The software developed to run in the smart-watch is an Android Wear application. In order to get access to the state of the WiFi signal, some permissions were switched on, including those needed to automatically start the application when the smart-watch is switched on.

The MQTT client library used for communicating with the back-end system were Apache Paho [44], which can be used as an Android service. Among other characteristics, Apache Paho supports MQTT version 3.1, SSL/TLS for secure connections and automatic reconnect. On the one hand, functional programming in Java is available form version 8, on the other hand, Android SDK uses Java version 7, in order to take advantage of the functional programming paradigm the Retrolambda [45] library was used, this way the code is easy to write, read and maintain.

Direct connection between the smart-watch and a know WAP can be lost. In this case, the samples taken can not be transmitted using the Apache Paho MQTT client, so these samples are internally stored using the SQLite database engine [46]. To ease the development the OrmLite Object Relational Mapping [47] was used to map Java objects to SQL tables.

### 3.4. Back-End Architecture

The back-end system is composed of two different parts: the IoT broker, the Machine Learning infrastructures, and the web-base configuration interface. The IoT broker is in charge of maintaining the communication between each client and the broker; the Machine Learning infrastructure is in charge of creating the classifiers needed to location each user indoors. Finally, the Web-based configuration interface is in charge of managing the users, the smart-watches devices and the links between them.

#### 3.4.1. IoT Broker and Machine Learning Infrastructures

The communication between a smart-watch and the back-end uses the MQTT connectivity protocol. The MQTT broker used was Apache Apollo [48], which is an Open Source multi-protocol broker, including the MQTT connectivity protocol. The broker is in charge of serving the messages between the publishers and the subscribers according to the QoS defined by the subscriber when connected to the broker.

During the configuration phase, the Signal Strength of all visible WAPs for the current location are used to build up a Machine Learning classifier, which later will provide an estimate for the current location based on the WiFi Signal Strength sampled. The library used to build the classifiers was Weka [49]. The Weka library provides most of the Machine Learning algorithm, in particular the following classifiers were used in this work: Multilayer Perceptron, Support Vector Machine, Decision Tree, Random Forest and Bayesian Network.

All samples, including those used to build up the classifiers, but also those used to locate the user, are stored in an NoSQL Elasticsearch [50] database. This database engine was chosen because it is flexible enough to include new fields, such as sensor readings, free text messages, and so on. In the case of including text messages, Elasticsearch provides a powerful search engine on top of the Lucene [51] library.

#### 3.4.2. Web-Based Configuration Interface

**Figure 3:**
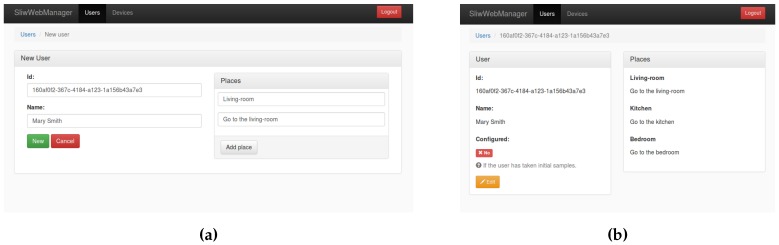
Process for registering a new user. (**a**) Setting up a new user; (**b**) New user created.

**Figure 4:**
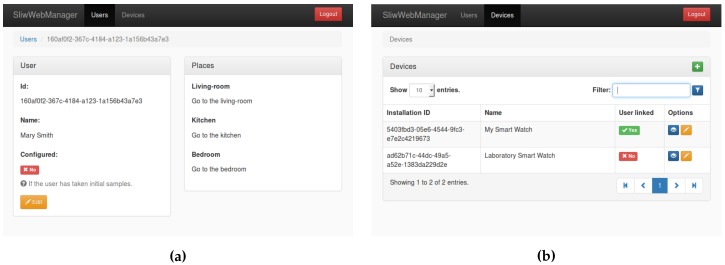
Devices management. (**a**) A name is given to the new device registered; (**b**) List of all devices registered.

**Figure 5:**
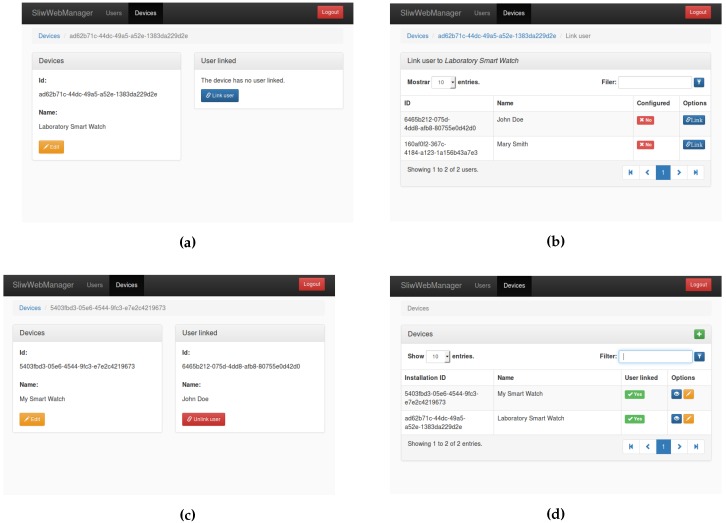
Process for linking a device to a user. (**a**) Setting up a new link; (**b**) Selecting a user to be linked to a device; (**c**) User already linked; (**d**) Device showing that a user is linked to it.

In order to ease the management of the users, devices and the operations related with them, a web application was developed. This application was coded in HTML and javascript. It interacts with the Elasticsearch database engine to manage users and devices, using a web interface (see Figure 3, Figure 4 and Figure 5). On the one hand, each new user is registered through the web configuration interface, on the other hand, each new device is automatically registered through a configuration step. Once a device is linked to an existing user, the creation of the classifiers starts (see Section 4 for details).

The technologies used to develop the web-based configuration interface were Bootstrap to develop the user interface and jquery for making AJAX requests.

### 3.5. Deployment

From previous section it can be seen that the complete system is composed of several collaborative sub-systems. In order to ease the deploy of all these sub-systems docker containers [52] were used. In particular five different docker images were created:

- ElasticSearch: this image is in charge of deploying the Elasticsearch database engine.
- ApacheApollo: this image is in charge of deploying the Apache Apollo MQTT broker.
- *AwareIndoorLoc* webmanager: this image is in charge of deploying the web-based application to manage the users, devices and the links between them.
- *AwareIndoorLoc* server: this image is in charge of deploying the Machine Learning algorithms.
- Kibana: This is an optional image, used to monitor and consult the Elasticsearch databases.

Finally, docker-compose is used to deploy all services using just one command.

## 4. System Operation

This section describes how the system operates. First, user and device management is described. Second, the procedure to make a link between a user and a device is presented. Then, it is described how the classifiers are built based on the WiFi signal strengths. Finally, the background process that runs in the smart-watch device to provide the signal strengths for the current location is described.

### 4.1. User Management

Figure 3 shows the web-based interface used to register a new user in the system. When registering a new user, the list of places where samples will be taken is created. Each place is accompanied with a descriptive text which will be shown to the user in the smart-watch window when acquiring the sample data to build the Machine Learning classifiers (see Section 4.4).

### 4.2. Device Management

Each device is automatically registered when the android wear application runs for the first time. Using the web-based application (see Figure 4) the list of all registered devices can be managed, in particular, a device can be linked to an existing user. Each device is identified by a number which is automatically generated when the Android Wear application is installed in the device, this way, the same physical device can be re-used just by re-installing the application on it.

### 4.3. Link Between User and Device

Once a new device is registered, it can be linked to any of the existing users. Figure 5 shows how to link a device to a user using the web-based configuration interface. At the end of the process the device will appear as linked. At this point, the device will be ready for downloading the places configured for the user in the previous step. This information will be used to obtain the WiFi signal strengths needed to create a WiFi mapping. At the end of this phase the device will be ready for sampling the WiFi signal strengths, and the Machine Learning classifiers used to locate the user indoors.

### 4.4. WiFi Mapping and Classifiers Building

**Figure 6:**
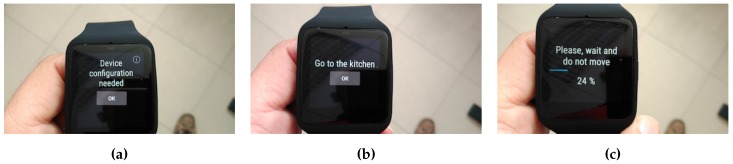
Some procedure steps to map the environment and create the Machine Learning classifiers. (**a**) Configuration starting; (**b**) User asked to go to the kitchen; (**c**) Sampling WiFi signal strength.

The first step before building the classifiers is to acquire the database with the WiFi signal strengths for each configured location. This step is needed to be performed just once, with a minimum intervention of the user, the only intervention needed is to push a button in the smart-watch for each location to be mapped. In this acquisition phase, the user is asked to go to every place and, once there, to start the acquisition for that place (see Figure 6). To acquire the samples is a time consuming task [36], so the number of samples taken have been balanced with the acquisition time needed trying to minimize the users fatigue, and maintaining a hit rate enough good for the system to be useful. Empirically, and for all scenarios tested, we needed one minute on average to take 50 samples. Depending on the particular scenario, and for four different locations, the time devoted to this step ranged between 10 and 15 min in total. The samples were taken for each location and sent to the server using the MQTT connectivity protocol. In case there was no connection to any known WAP, the samples are stored locally on the device and will be sent when a connection will be available. On average, an for the smart-watch used in this work, less than one minute is enough to sample and transmit the results. The same process is repeated until all configured locations are mapped.

Once all mapping data is available, the next step is to build the Machine Learning classifiers presented in Section 3.4.1. This step is ran in the server and the five classifiers built are serialized and stored in the Elasticsearch database, this way, if the server goes down, all classifiers can be recovered. Once the classifiers are built, the smart-watch device is notified to start running a background process which periodically sends the signal strengths samples to the server.

### 4.5. Background Process

A background process running in the wearable device was configured to wake up every minute, sample the WiFi signal strengths around and send them to the server. These samples are used to find out the current location of the user and, after that, the samples and the location are stored in the Elastisearch database. If there was no connection with the server, the samples are stored locally in the device and sent when an available connection is detected.

Optionally, the user can perform two more operations. He or she can get a new sample in order to improve the accuracy of the classifiers if needed, selecting the current location where the new sample is taken. Using this new sample and all previous ones, the classifiers are re-built. The second optional operation is used to test the location accuracy of the system; in this case a sample is taken and sent to the server which, in turn, will return the estimated location provided by the classifiers. This background process is started in the smart-watch device every time it is booted.

## 5. Performance Evaluation through Real Experiments

This section presents the characteristics of the scenarios used for experimentation; then, the characteristics of the WiFi fingerprinting databases obtained are described; finally, the experimental results are presented.

### 5.1. Experimentation Scenarios Description

All scenarios used during the experimentation phase were urban flats, three of them located in the city of Castellón de la Plana and the other two in the city of Valencia. Flats located in the same city are far enough to each other to not share any common WAPs. The size of the flats ranges between 120 m${}^{2}$ for the biggest one and 62 m${}^{2}$ for the smallest one. Different rooms can be used to build the fingerprinting database depending on the particular flat, Table 2 shows the main characteristics of the scenarios, and the selected rooms sampled in the experiments. The number of WAPs in Table 2 is the total number of WAPs present in all samples acquired in the locations mapped for each scenario. These samples include training and test steps. The number of WAPs include the WiFi network deployed in the user’s flat, and the WiFi networks deployed in neighbors’ flats too. These numbers are in accordance with the data published in reference [53].

**Figure 7:**
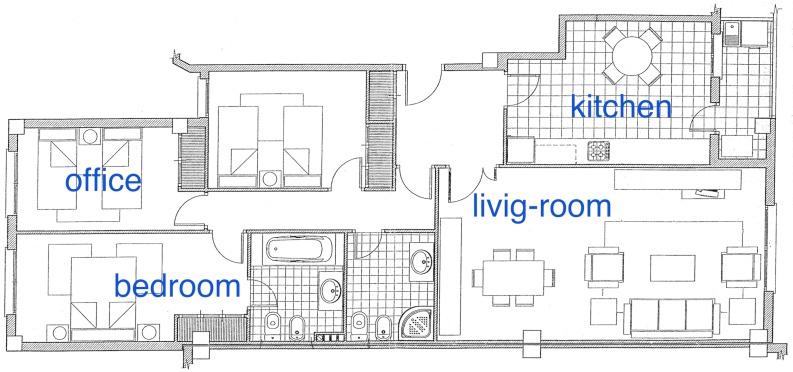
Scenario number 1 used in the experiments.

Figure 7 shows the house plan for Scenario 1. The legends show the rooms used in the sampling process. Note that the office is by the bedroom, and also the living-room is by the kitchen, but each pair is relatively far from the other.

### 5.2. Characteristics of the Obtained Databases

Four different databasea were created for each scenario: the first two were used to build the five different Machine Learning classifiers (training database), the second two were used to validate the performance of those classifiers (testing database). Each training database was composed of 50 different samples for each location defined in the scenario. Each testing database was composed of 100 samples for each location. The difference between the two training databases was that the 50 samples for each location were acquired with the user standing up at the same position in the room, and in the other case the user was moving around the room. The standing up samples try to mimic when the user remains at the same place for a period of time, for example when the user is watching television sited in his/her sofa. Before taking the measures we ask the users to use their preferred locations. The moving around samples try to mimic the user’s behavior when moving within a room or moving between different rooms. The two testing databases were collected in the same way.

### 5.3. Setup and Experimental Results

The five selected classification algorithms were tested for each training database against each testing database, given four different sets of results for each scenario. In order to test if there was any improvement on using the results provided for more than one classifier, an ensemble classifier was also built with the results of all five classifiers based on a voting scheme. Each classifier emits a vote which is its estimation for the user’s location, so if two classifiers estimate the same location for the user, for example Kitchen, this location receives two votes, the final location provided by the ensemble is that with the highest vote rate. Also, a second classifier (Ensemble (2)) was built with the two classification algorithms that provide the best hit rate on average for the five scenarios, namely Random Forest and Bayes Network. To avoid ties in the case, the final location provided by the ensemble is that with the minimum uncertainty provided by each of the two classifiers. Table 3 shows the performance results obtained for all seven classifiers at each scenario, and the four different experiments. The average values were obtained averaging the results for the five different scenarios tested.

Regarding the average results provided by the ensemble classifier, the strategy which provided the highest hit rate was standing up for training and standing up for testing, followed by moving for training and moving for testing. These two strategies also performed the best, on average, for the five classifiers used.

Regarding the average results provided by each classifier, the highest hit rate was provided by the Bayes Network classifier for all different strategies, followed by the Random Forest algorithm. The Bayes Network classification algorithm provides the best hit rate in 13 of the 20 experiments performed for this classifier, followed by Multilayer Perceptron which provides the best result in 3 of 20 experiments, but remarkably these experiments were performed in the same scenario.

Regarding the same scenario, Bayes Network gave the highest hit rate for three of the strategies used, with a minimum difference regarding Random Forest the standing up for training moving for testing, for the first scenario. Again, in the second, third and fourth scenario the Bayes Network classifier provided the highest hit rate for three of the four strategies used. The only scenario which gave different results was the fifth one where the Multilayer Perceptron gave the highest results for three of the four strategies used.

For all scenarios tested, there was one strategy, at least, that gave a hit rate greater than 80% for the ensemble classifier. Also, for all scenarios tested, and taking into account the results individually provided by the five classifiers, there was one strategy, at least, that gave a hit rate greater than 87%. In particular, Scenario 1 provided 100% hit rate for the Bayes Network classifier. In the case of the Ensemble (2) classifier, the only scenario with a hit rate below 80% for all strategies was the fifth one, which gave a maximum hit rate for the moving for training and moving for testing strategy (73.50%).

An ensemble using the Bayes Network and Random Forest was tested too. These two algorithms were the first and second classification algorithm with the highest hit rate on average for the five scenarios tested, respectively. In the cases of the standing up for training and standing up for testing, and moving for training and standing up for testing strategies there was an increase in the hit rate. In contrast, the hit rate decreased for the other two strategies, with a minimum difference in the case of the standing up for training and moving for testing.

Table 4, Table 5, Table 6 and Table 7 show the confusion matrices for the first scenario, the four different experiments performed for the five classifiers and the ensemble built with the voter coming from the five classifiers. Confusion matrices for the four other scenarios provided a qualitative result quite similar to the first scenario.

Confusion matrices in all experiments show that there is a trend to confuse those rooms close to each other. For Scenario 1, the living-room is by the kitchen, and also the bedroom is by the office, and on the opposite, these last are relatively far from the living-room and the kitchen. So, the classification algorithms tends to miss-classify these rooms. Similar results remain for the other Scenarios tested.

### 5.4. Battery Life Estimation

One major concern when using wearable devices is battery life, specially when WiFi connection is intensively used. To estimate battery life of the smart-watch two experiments were performed. In the first experiment, the user in Scenario 1 was provided with two smart-watches of the same model, and asked to wear them during six days. The Android wear application was installed in one of the smart-watches and the WiFi connection was switched on. The other smart-watch acted as control, the WiFi connection was switched off. Bluetooth connection was active in both smart-watches. Both smart-watches were recharged at night and remained plugged to an electric outlet all night long. Before plugging, the battery charge on both smart-watches was registered. Both smart-watches were unplugged when the subject woke up.

In the second experiment we estimate how battery life depends on the interval between WiFi measures. Three different interval between measures were defined 60, 120 and 300 s, and the same operational procedure than in the first experiment was used, namely the user was asked to wear both smart-watches. Table 8 summarizes the results obtained.

The first conclusion is that continuous use of WiFi dramatically drains battery life, although one charge is enough for awake time period of a day. The second conclusion is that battery life increases when time between measures increases too. This way battery life and time between measurements can be balanced to record enough samples without draining battery life.

## 6. Discussion

The system presented gave a hit rate greater than 80% for the best strategy for all tested scenarios in the case of the ensemble classifier. If the results provided by the classifiers were individually taken, this percentage increased up to 87%, which could be considered an accuracy good enough to be used as a positioning algorithm. Therefore, this system would be feasible as a base positioning technology for developing an AAL application on top of it.

The strategy chosen to position a user dramatically depends on the type of behavior at his/her home: if the user remains standing up in each room most of the time (watching TV sitting in a coach in front of it in the living-room, having lunch sitting in a table in the kitchen, sleeping in the bedroom, and so on), the most convenient strategy to take samples for building the classifiers is standing up in each room. In contrast, if the user moves around each room, the most convenient strategy to take the samples will be with the user moving around at each room.

The sampling strategy which provides the greatest hit rate for the ensemble classifier on average and for all scenarios tested was to take samples standing up for training, and to take samples standing up for testing too.

When sorting the five tested classifiers by its hit rate (see Table 3), and taking into account the results for all scenarios tested, we obtain: Bayes Network, Random Forest, Multilayer Perceptron, Support vector Machines and C4.5.

In the case of the ensemble classifier, there was not an improvement in the hit rate when using the classifiers which gave the highest hit rate. To choose a convenient ensemble dramatically depends on the real scenario.

Confusion matrices show the difficulty distinguishing rooms close to each other. One possible solution to minimize this issue could be to take more samples for adjoining rooms. Another possibility could be to better analyze the fingerprints for adjacent rooms.

All WiFi signal strengths will be used when building the classifiers, independently of its signal strength level. It would be interesting to use only those signal strengths greater than some threshold, in order to remove the signals with low intensity.

Battery life is a major concern regarding wearable devices. Although the use of WiFi dramatically drains battery life, it has been empirically tested that one charge is enough for using WiFi when the user is awake. Promising new Passive WiFi technologies [54] could dramatically mitigate battery life for wearable devices in the short term.

## 7. Conclusions and Future Work

In this paper, we have presented an indoor WiFi fingerprinting positioning system based on wearable devices and Machine Learning algorithms. The system is robust, reliable, secure and unobtrusive, facilitating its use at home. The selected wearable device is a smart-watch running an Android Wear application that periodically samples WiFi signal strengths and sends them to a server to estimate the current location of a user. Communication between the wearable device and the server were performed using the MQTT connectivity protocol. To use a smart-watch is a convenient solution because it can be comfortably worn and it is linked to the user as he moves around his home. In the experiments performed, one single battery charge was enough to run the device for a whole day.

Based on the samples taken in the configuration step, five different Machine Learning classifiers were built. An ensemble classifier was built using the votes coming from the five classifiers: each classifier emits a vote to classify every new sample and the final location is taken as the best estimate for the current location of the user. For the ensemble classifier (see Table 3), the experiments performed show that the hit rate when classifying a user is very high (92.00% in the best case, 71.07% on average for all scenarios and for all experiments performed, Ensemble column on Table 3).

Experiments were performed to empirically test battery life during hours spent awake, and the results showed that it is possible to balance battery life and time between measurements in order to not drain battery life.

As future work we have planned to add to each message sent to the server the readings of all existing sensors in the device, this information can be used for specific uses such as fall detection; a fall could be more dangerous if it occurs in the bathroom than in the living-room. Another useful extension of our work would be to determine if a user has exited his home, in such a case, the GPS location could be switched on to locate the user outdoors. In contrast, the GPS could be switched off if the user arrives at home, saving battery life and starting indoor positioning by means of the WiFi signal strengths.

Finally, our system could be easily integrated with other smart-home systems such as electricity consumption: if the system detects that the user is out of a room for a pre-determined period of time, the lights in that room could be switched off.

## Figures and Tables

**Table 1:** In home monitoring systems comparison.

Cite	*Sensor Type*	*Cost*	*Scalability*	*Obtrusive*	*Connection*	*Interoperable*	*Extensibility*
[23]	IR, magnetic switches & ad-hoc sensor	Medium	Medium	Medium	Not specified	Yes	Yes
[24]	IR, magnetic, body constants	Average	Medium	Ethical issues	CAN	Data level (XML)	Yes
[33]	Wearable, environmental and cameras	Expensive	Medium	Medium	Wireless	Yes	Yes
[26]	RFID card	Average	High	Medium	RFID	Yes	No
[34]	Wearable camera, microphones and sensors	Expensive	High	High	ZigBee	Yes	Yes
[35]	Wearable camera	Expensive	High	High	Not specified	No	Yes
[27]	Capacitive sensors	Average	Low	Medium	USB	No	No
[36]	WiFi	Cheap	High	Low	Mobile phone	Yes	Yes
[29]	Badges	Average	Medium	Low	Ultrasounds	Yes	Yes
[30]	Beacons and transponders	Expensive	Medium	Low	Microwave signals	Yes	Yes
[31]	Beacons	Not provided	High	Low	Bluetooth	Proprietary	Yes
[28]	Zigbee sensors	Expensive	Medium	Low	Zigbee	Yes	Yes
[32]	IR	Not provided	High	Medium	Not specified	Yes	Yes
Ours	WiFi	Cheap	High	Low	WiFi	Yes	Yes

**Table 2:** Scenario characteristics, the size for each flat is given in squared meter. The total number of WAPs states for the total number of WAPs present in all samples acquired in the experiments for each scenario.

Scenario	Size	Total Number of WAPs	Locations Mapped
1	120	33	Kitchen, Office, Living-room, Bedroom
2	80	36	Kitchen, Office, Living-room, Bathroom
3	90	27	Kitchen, Office, Living-room, Bedroom
4	80	43	Kitchen, Office, Living-room, Bedroom
5	62	23	Kitchen, Office, Living-room, Bedroom

**Table 3:** Performance results for the five different Machine Learning classifiers (data shown in percentages. Samples for training acquired static samples for testing too. The best result for each scenario is shown in bold.

Scenario	Perceptron	SVM	C4.5	Random Forest	Bayes Net	Ensemble	Ensemble(2)
**Standing up Training Standing up Test**							
1	78.50 ± 0.30	78.50 ± 0.34	64.00 ± 0.42	84.50 ± 0.22	**100.00** ${}_{-0.01}^{+0.00}$	83.75 ± 0.33	100.00 ± 0.11
2	82.50 ± 0.25	78.00 ± 0.35	**94.00 ± 0.17**	92.50 ± 0.25	85.50 ± 0.24	92.00 ± 0.29	80.50 ± 0.18
3	60.75 ± 0.40	53.00 ± 0.39	49.75 ± 0.49	55.50 ± 0.38	**71.00** ± 0.37	59.25 ± 0.46	85.00 ± 0.26
4	64.25 ± 0.38	74.75 ± 0.35	80.00 ± 0.32	83.00 ± 0.26	**90.50 ± 0.21**	82.00 ± 0.35	97.50 ± 0.28
5	**89.75 ± 0.19**	80.75 ± 0.34	47.75 ± 0.51	75.50 ± 0.32	56.75 ± 0.43	80.25 ± 0.42	69.75 ± 0.27
Average	75.15 ± 1.52	73.00 ± 1.77	67.10 ± 1.91	78.20 ± 1.43	**80.75 ± 1.26**	79.60 ± 1.85	86.55 ± 1.24
**Standing up Training Moving Test**							
1	70.50 ± 0.37	68.75 ± 0.35	54.75 ± 0.47	**80.75 ± 0.27**	80.00 ± 0.30	72.50 ± 0.40	85.75 ± 0.20
2	62.25 ± 0.36	64.50 ± 0.37	73.25 ± 0.36	80.75 ± 0.28	**92.00 ± 0.19**	76.75 ± 0.36	90.00 ± 0.17
3	51.00 ± 0.43	61.00 ± 0.38	43.50 ± 0.53	56.50 ± 0.38	**62.25 ± 0.41**	59.50 ± 0.41	70.50 ± 0.28
4	78.00 ± 0.30	74.75 ± 0.34	76.25 ± 0.35	**92.50 ± 0.21**	85.25 ± 0.26	87.25 ± 0.26	46.75 ± 0.29
5	**73.25 ± 0.32**	66.00 ± 0.38	36.50 ± 0.56	57.75 ± 0.37	54.75 ± 0.44	58.50 ± 0.47	55.25 ± 0.29
Average	67.00 ± 1.78	67.00 ± 1.82	56.85 ± 2.27	73.65 ± 1.51	**74.85 ± 1.60**	70.90 ± 1.90	69.65 ± 1.49
**Moving Training Moving Test**							
1	75.50 ± 0.33	75.75 ± 0.35	76.50 ± 0.34	85.50 ± 0.24	**88.50 ± 0.21**	79.50 ± 0.34	59.25 ± 0.16
2	45.50 ± 0.48	43.25 ± 0.40	53.00 ± 0.48	52.75 ± 0.36	**77.50 ± 0.31**	52.25 ± 0.46	59.75 ± 0.24
3	78.25 ± 0.28	81.75 ± 0.34	72.25 ± 0.37	85.75 ± 0.24	**87.00 ± 0.22**	86.75 ± 0.33	85.25 ± 0.16
4	83.00 ± 0.26	82.50 ± 0.34	78.75 ± 0.31	92.50 ± 0.20	**92.50 ± 0.18**	91.25 ± 0.29	50.50 ± 0.13
5	70.25 ± 0.38	58.25 ± 0.36	56.75 ± 0.46	64.25 ± 0.33	**73.25 ± 0.34**	61.50 ± 0.42	73.50 ± 0.23
Average	70.10 ± 1.73	68.30 ± 1.79	67.45 ± 1.96	76.15 ± 1.37	**83.75 ± 1.26**	74.25 ± 1.84	65.65 ± 1.61
**Moving Training Standing up Test**							
1	78.50 ± 0.31	80.25 ± 0.34	80.75 ± 0.31	96.50 ± 0.23	**99.00 ± 0.06**	84.75 ± 0.30	91.25 ± 0.12
2	38.50 ± 0.51	29.75 ± 0.42	46.00 ± 0.52	45.75 ± 0.40	**49.00 ± 0.49**	33.25 ± 0.53	84.75 ± 0.32
3	58.75 ± 0.43	**59.75 ± 0.40**	52.50 ± 0.48	51.75 ± 0.39	57.50 ± 0.44	60.00 ± 0.48	92.75 ± 0.29
4	66.50 ± 0.40	66.25 ± 0.37	59.50 ± 0.45	73.50 ± 0.29	**76.00 ± 0.32**	70.50 ± 0.41	81.75 ± 0.21
5	**63.00 ± 0.39**	39.50 ± 0.39	37.25 ± 0.56	51.00 ± 0.36	53.50 ± 0.43	49.25 ± 0.48	52.75 ± 0.28
Average	61.50 ± 2.04	55.10 ± 1.92	55.20 ± 2.32	63.70 ± 1.67	**67.37 ± 1.74**	59.55 ± 2.20	80.65 ± 1.57

**Table 4:** Confusion matrix for the first scenario. The samples for training were taken with the user standing up, and also standing up for testing. The meaning of the capital letters are: L: Living-room, K: Kitchen, O: Office, B: Bathroom.

	Multilayer Perceptron				SVM				C4.5			
	L	K	O	B	L	K	O	B	L	K	O	B
L	84	16	0	0	84	16	0	0	99	1	0	0
K	0	100	0	0	0	100	0	0	28	72	0	0
O	0	1	30	69	0	1	30	69	0	73	16	11
B	0	0	0	100	0	0	0	100	0	31	0	69
	**Random Forest**				**Bayes Network**				**Ensemble**			
	L	K	O	B	L	K	O	B	L	K	O	B
L	100	0	0	0	100	0	0	0	99	1	0	0
K	0	100	0	0	0	100	0	0	0	100	0	0
O	0	1	38	61	0	0	100	0	0	1	36	63
B	0	0	0	100	0	0	0	100	0	0	0	100

**Table 5:** Confusion matrix for the first scenario. The samples for training were taken with the user standing up, and moving for testing. The meaning of the capital letters are: L: Living-room, K: Kitchen, O: Office, B: Bathroom.

	Multilayer Perceptron				SVM				C4.5			
	L	K	O	B	L	K	O	B	L	K	O	B
L	69	31	0	0	69	31	0	0	89	11	0	0
K	1	99	0	0	1	99	0	0	39	61	0	0
O	0	1	14	85	0	2	15	83	0	12	15	73
B	0	0	0	100	0	8	0	92	0	46	0	54
	**Random Forest**				**Bayes Network**				**Ensemble**			
	L	K	O	B	L	K	O	B	L	K	O	B
L	74	26	0	0	57	43	0	0	73	27	0	0
K	1	99	0	0	1	99	0	0	1	99	0	0
O	0	1	50	49	0	1	84	15	0	1	20	79
B	0	0	0	100	0	3	17	80	0	2	0	98

**Table 6:** Confusion matrix for the first scenario. The samples for training were taken with the user moving, and also moving for testing. The meaning of the capital letters are: L: Living-room, K: Kitchen, O: Office, B: Bathroom.

	Multilayer Perceptron				SVM				C4.5			
	L	K	O	B	L	K	O	B	L	K	O	B
L	90	8	2	0	83	17	0	0	100	0	0	0
K	1	95	4	0	1	99	0	0	15	85	0	0
O	0	1	97	2	0	1	97	2	2	0	93	5
B	0	0	80	20	0	0	76	24	34	0	38	28
	**Random Forest**				**Bayes Network**				**Ensemble**			
	L	K	O	B	L	K	O	B	L	K	O	B
L	99	1	0	0	98	2	0	0	98	2	0	0
K	3	97	0	0	1	99	0	0	1	99	0	0
O	0	1	97	2	0	1	93	6	0	1	96	3
B	0	0	51	49	0	0	36	64	0	0	75	25

**Table 7:** Confusion matrix for the first scenario. The samples for training were taken with the user moving, and standing up for testing. The meaning of the capital letters are: L: Living-room, K: Kitchen, O: Office, B: Bathroom.

	Multilayer Perceptron				SVM				C4.5			
	L	K	O	B	L	K	O	B	L	K	O	B
L	86	12	2	0	84	16	0	0	100	0	0	0
K	0	95	5	0	0	100	0	0	10	90	0	0
O	0	0	85	15	0	0	85	15	0	0	85	15
B	0	0	52	48	0	0	48	52	33	0	19	48
	**Random Forest**				**Bayes Network**				**Ensemble**			
	L	K	O	B	L	K	O	B	L	K	O	B
L	97	3	0	0	96	4	0	0	98	2	0	0
K	5	95	0	0	0	100	0	0	5	95	0	0
O	0	0	100	0	0	0	100	0	0	0	85	15
B	0	0	6	94	0	0	0	100	0	0	39	61

**Table 8:** Battery usage for a smart-watch running the indoor location Android application compared with a smart-watch which does not run the application. The time between measures is shown in seconds. The charge remaining is shown as a percentage of the full charge.

Day	Time Interval	Time between Measures	Battery Charge Located	Battery Charge no-Located
1	7:30–22:00	60	16%	87%
2	8:30–22:30	60	29%	90%
3	8:00–21:30	60	5%	75%
4	8:00–21:30	120	20%	75%
5	8:00–21:30	120	24%	74%
6	8:00–21:30	300	40%	67%
7	8:00–21:30	300	41%	74%
